# Cigarette consumption from a life-course perspective in low- and middle-income countries

**DOI:** 10.2471/BLT.24.292918

**Published:** 2025-07-09

**Authors:** Mark Goodchild, Jeremias Paul, Ruediger Krech

**Affiliations:** a Health Promotion Department, World Health Organization, 20 Avenue Appia, 1211 Geneva, Switzerland.

## Abstract

**Objective:**

To calculate the total life-course expenditure of smokers on cigarettes alone, before or without accounting for any economic losses as a result of smoking-attributable death and disease.

**Method:**

We used data from Global Adult Tobacco Surveys to calculate annual cigarette consumption and expenditure in 15 low- and middle-income countries. We extracted data on average earnings from the ILOSTAT database of the International Labour Organization. We calculated life-course cigarette expenditures using cohort life expectancies and inflation, and converted these expenditures into net present value terms using a 3% social discount rate.

**Findings:**

The average age of adult cigarette smokers in our sample was 40 years, and their average expenditure on cigarettes was equivalent to 7.2% of annual average earnings. Given an average life expectancy of 55 years at the age of 15 years, we estimated an average life-course consumption of 217 752 cigarettes and a full life-course expenditure of 8481 United States dollars (US$) in net present value terms, more than twice the current average annual earnings of workers. However, by quitting, current adult smokers can avoid an average of US$ 6612 in expenditure on cigarettes over their remaining life-course.

**Conclusion:**

The affordability of cigarettes is an important determinant of cigarette use and tax policies can have a large effect on consumers, especially young adults. These costs will only increase over time as governments continue to raise taxes to address the market failures inherent within the tobacco market.

## Résumé

**Objectif:**

Calculer les dépenses totales consacrées par les fumeurs à l’achat de cigarettes au cours de leur vie, avant de tenir compte ou sans tenir compte des pertes économiques liées au décès et aux maladies attribuables au tabagisme.

**Méthode:**

Nous avons utilisé les données des enquêtes mondiales sur le tabagisme chez les adultes (Global Adult Tobacco Survey, GATS) pour calculer la consommation annuelle de cigarettes et les dépenses correspondantes dans 15 pays à revenu faible et intermédiaire. Nous avons extrait de la base de données ILOSTAT de l’Organisation internationale du travail les données sur les revenus moyens. Nous avons calculé les dépenses liées à la consommation de cigarettes tout au long de la vie en utilisant l’espérance de vie des cohortes et l’inflation, puis nous avons converti ces dépenses en valeur actuelle nette en appliquant un taux d’actualisation social de 3%.

**Résultats:**

L’âge moyen des fumeurs adultes dans notre échantillon était de 40 ans, et leurs dépenses moyennes en cigarettes représentaient 7,2% de leur revenu annuel moyen. Compte tenu d’une espérance de vie moyenne de 55 ans lorsque les sujets sont âgés de 15 ans, nous avons estimé une consommation moyenne de 217 752 cigarettes au cours de la vie et une dépense totale de 8 481 dollars américains (USD) en valeur actuelle nette, ce qui représente plus du double du revenu annuel moyen actuel des travailleurs. Cependant, en arrêtant de fumer, les fumeurs adultes actuels peuvent éviter en moyenne 6 612 USD de dépenses en cigarettes au cours du reste de leur vie.

**Conclusion:**

Le caractère abordable des cigarettes constitue un facteur déterminant de leur consommation, et les politiques fiscales sont de nature à avoir un effet important sur les consommateurs, en particulier les jeunes adultes. Les coûts associés ne feront qu’augmenter parallèlement au relèvement des taxes par les pouvoirs publics en vue de remédier aux défaillances inhérentes au marché du tabac.

## Resumen

**Objetivo:**

Calcular el gasto total a lo largo del curso de vida de las personas fumadoras únicamente en cigarrillos, sin considerar las pérdidas económicas derivadas de enfermedades o muertes atribuibles al tabaquismo.

**Métodos:**

Se utilizaron datos de las encuestas globales sobre el tabaquismo en adultos para calcular el consumo y gasto anual en cigarrillos en 15 países de ingresos bajos y medios. Se extrajeron datos sobre los ingresos promedio de la base de datos ILOSTAT de la Organización Internacional del Trabajo. El gasto a lo largo del curso de vida se calculó utilizando las expectativas de vida por cohorte y ajustando por inflación, y se convirtió a términos de valor actual neto utilizando una tasa de descuento social del 3%.

**Resultados:**

La edad promedio de las personas adultas fumadoras en la muestra fue de 40 años y su gasto promedio en cigarrillos equivalía al 7,2% del ingreso anual promedio. Considerando una expectativa de vida promedio de 55 años a partir de los 15 años de edad, se estimó un consumo total a lo largo del curso de vida de 217 752 cigarrillos y un gasto acumulado de 8481 dólares estadounidenses (US$) en valor actual neto, más del doble del ingreso promedio anual actual de los trabajadores. No obstante, si abandonaran el consumo, las personas fumadoras adultas actuales podrían evitar, en promedio, un gasto de US$ 6612 en cigarrillos durante el resto de su vida.

**Conclusión:**

La asequibilidad de los cigarrillos es un factor determinante clave en su consumo. Además, las políticas fiscales pueden influir considerablemente en las decisiones de consumo, en especial entre la población adulta joven. Estos costos solo aumentarán con el tiempo, en la medida en que los gobiernos continúen incrementando los impuestos para corregir las fallas del mercado inherentes al mercado del tabaco.

## ملخص

الغرض حساب إجمالي إنفاق المدخنين على السجائر وحدها طوال حياتهم، قبل أو دون احتساب أي خسائر اقتصادية ناجمة عن الوفاة أو المرض المرتبطين بالتدخين.

الطريقة استخدمنا بيانات من المسوحات العالمية للتبغ لدى البالغين (Global Adult Tobacco Surveys) لحساب الاستهلاك السنوي للسجائر والإنفاق عليها في 15 دولة من الدول ذات الدخل المنخفض والدخل المتوسطة. قمنا باستخراج بيانات عن متوسط الدخل من قاعدة بيانات منظمة العمل الدولية (ILOSTAT). قمنا بحساب النفقات على السجائر طوال الحياة باستخدام متوسطات العمر المتوقع للفئة العمرية ومعدل التضخم، وحولنا هذه النفقات إلى صافي القيمة الحالية باستخدام معدل خصم اجتماعي قدره %3.

النتائج بلغ متوسط عمر المدخنين البالغين في العينة لدينا 40 عامًا، وكان متوسط إنفاقهم على السجائر يعادل %7.2 من متوسط الدخل السنوي. بالنظر إلى متوسط العمر المتوقع البالغ 55 عامًا عند سن 15 عامًا، فقد قدرنا أن متوسط استهلاك دورة الحياة بـ 217752 سيجارة، ونفقات دورة الحياة الكاملة بـ 8481 دولارًا أمريكيًا من حيث القيمة الحالية الصافية، أي أكثر من ضعف متوسط الدخل السنوي الحالي للعمال. ومع ذلك، ومن خلال الإقلاع عن التدخين، يمكن للمدخنين البالغين الحاليين تجنب متوسط 6612 دولارًا أمريكيًا من الإنفاق على السجائر خلال دورة حياتهم المتبقية.

النتائج تُعد القدرة على تحمل تكاليف السجائر عاملاً مهمًا في تحديد استخدام السجائر، ويمكن أن يكون للسياسات الضريبية تأثير كبير على المستهلكين، وخاصة الشباب الصغار. ستزداد هذه التكاليف فقط بمرور الوقت مع استمرار الحكومات في رفع الضرائب لمواجهة إخفاقات السوق المتأصلة في سوق التبغ.

## 摘要

**目的:**

旨在计算吸烟者生命全程中仅在香烟方面的总支出，不包含因吸烟所导致死亡和疾病造成的任何经济损失。

**方法:**

我们运用从全球成人烟草调查中获得的相关数据来计算 15 个中低收入国家的年度香烟消费量和支出。我们从国际劳工组织的 ILOSTAT 数据库中提取了与平均收入有关的数据。我们运用队列预期寿命和通货膨胀相关数据计算了生命全程的香烟支出，并采用 3% 的社会折现率将此类支出转化为净现值。

**结果:**

我们所抽取样本中成年吸烟者的平均年龄为 40 岁，且其在香烟方面的平均支出相当于年平均收入的 7.2%。假设吸烟者 15 岁时的平均预期剩余寿命为 55 年，我们估计其生命全程的平均香烟消费量为 217,752 根，且按净现值计算的生命全程支出总额为 8,481 美元 (US$)，是工人当前平均年收入的两倍以上。然而，在剩余生命全程中，当前成年吸烟者可通过戒烟避免平均金额为 6,612 美元的香烟支出。

**结论:**

香烟承付能力是影响香烟消费的一个重要决定性因素，且税务政策会对消费者（特别是年轻人）产生显著影响。随着时间的推移，此类成本只会逐渐上涨，因为各国政府会继续提高税收以解决烟草市场固有的市场失灵问题。

## Резюме

**Цель:**

Рассчитать затраты, которые курильщики несут на протяжении жизни, непосредственно на сигареты без учета или до вычетов любых иных экономических потерь вследствие обусловленных курением случаев смерти и заболеваний.

**Методы:**

Авторы использовали данные Глобального опроса взрослого населения о потреблении табака для расчета объема ежегодного потребления сигарет и связанных с этим затрат в 15 странах с низким и средним уровнем дохода. Данные о среднем уровне дохода были взяты авторами из базы данных ILOSTAT Международной организации труда. Расчет затрат на потребление сигарет на протяжении жизни проводился с учетом когортной ожидаемой продолжительности жизни и инфляции, и затем эти значения были приведены к чистой текущей стоимости с применением социальной ставки дисконтирования в 3% годовых.

**Результаты:**

Средний возраст взрослого курильщика сигарет в исследуемой выборке составил 40 лет, и средний уровень их расходов на сигареты был эквивалентен 7,2% от среднегодового дохода. Исходя из средней ожидаемой продолжительности жизни в 55 лет для 15-летних, авторы рассчитали, что на протяжении жизни среднее потребление сигарет составляет 217 752 штуки с соответствующими затратами в 8481 доллар США (US$) в пересчете на чистую текущую стоимость. Эта сумма более чем вдвое превышает нынешний среднегодовой заработок рабочего класса. Однако, отказавшись от сигарет, взрослые курильщики могут сэкономить в среднем 6612 долларов США, которые они в противном случае потратили бы на сигареты на протяжении оставшегося ожидаемого срока жизни.

**Вывод:**

Доступность сигарет является важной детерминантой их использования, и налоговая политика оказывает сильное влияние на потребителей, особенно на молодых людей. Эти затраты со временем будут только возрастать, поскольку правительства продолжают повышать налоги, чтобы справиться с проблемами, присущими рынку табака и табачных изделий.

## Introduction

A key justification for government interventions in the tobacco market, aside from preventing many unnecessary deaths and diseases, is that the consumption of tobacco products reflects several market failures. Many provisions of the World Health Organization (WHO) Framework Convention on Tobacco Control (FCTC) and the MPOWER package of tobacco control interventions seek to address these market failures, known as externalities and internalities. An externality is a cost borne by someone other than the smoker themselves, and an internality is a cost that smokers do not consider when deciding to use tobacco. These costs are not fully reflected in the market price of tobacco products, but may be hidden in the sense that the individual is not aware of or fully informed about the potential consequences of long-term use.

Another key internality is what is known in behavioural economics as hyperbolic discounting, whereby the immediate consequences of decisions are disproportionately favoured relative to any future consequences. Consumer preferences may also be inconsistent over time as people age,^1^ which helps to explain why, for example, surveys often find that adult smokers want to quit and regret taking up smoking. Behavioural economics are perhaps the most insightful when exploring the initiation of smoking in young adults, where the classical economic concept of the consumer as a rational and sovereign decision-maker is arguably the most questionable. Myopic forms of decision-making, in which future consequences are completely discounted, might initially prevail. Young adults are also potentially more open to experimentation and susceptible to peer influence. Not surprisingly, the price of cigarettes has been found to be a significant predictor of smoking experimentation among this age group.^2^

The long-term consequences of tobacco use can include death and disease as well as economic and financial impacts at the household level.^3^ There is a large body of evidence on the high and catastrophic (impoverishing) impact of expenditure to treat diseases resulting from tobacco use.^4^^–^^7^ Similarly, such diseases can cause absenteeism and presenteeism at work, as well as loss of household income. There is also strong evidence of everyday expenditure on tobacco products themselves having a diversionary effect, often in the form of less household spending on health care and education.^8^^–^^12^ The latest 2023–2024 Household Consumption Expenditure Survey in India, for example, includes the alarming statistic that rural households spent more on tobacco products than on education services.^13^ Because education presents a clear pathway towards breaking the cycle of generational poverty, such statistics undoubtedly reflect an unfortunate misallocation of household resources.

Recently published studies have shown increased interest in quitting since the coronavirus disease 2019 (COVID-19) pandemic, although there have also been relapses among former smokers.^14^^–^^18^ This somewhat idiosyncratic response to the pandemic may reflect a previous lack of awareness of the risks of smoking; more recent surveys have reported that smokers have included risks associated with COVID-19 in their reasons for quitting.^19^ These different responses also share some parallels with smoking behaviour during economic downturns.^20^^–^^22^ More broadly, we might expect increased public interest in the financial cost associated with tobacco use.

In this paper we therefore aim to calculate the cost to smokers over their life-course simply from everyday spending on cigarettes, without even beginning to account for any economic costs caused by smoking-attributable death and disease. These estimates are particularly relevant to young adults who may not fully appreciate the long-term financial implications of addiction. We also highlight the large savings (avoided expenditure) available to current adult smokers who quit tobacco, even in later years of life. Finally, we discuss the effect of several policies of the MPOWER package, namely taxation and cessation support, on successful cessation.^23^^,^^24^

## Methods

We extracted data on smoking behaviour from the Global Adult Tobacco Survey (GATS) conducted during the past decade in 15 countries;^25^ these particular countries account for almost two thirds of smokers in all low- and middle-income countries.^26^^,^^27^ We sourced epidemiological data on cohort life expectancy from WHO life tables for 2020,^28^ and data on average annual earnings of employees from the ILOSTAT database of the International Labour Organization.^29^ Lastly, we used a projected rate of inflation for emerging market and developing economies obtained from the April 2024 World Economic Outlook Database of the International Monetary Fund.^30^

We calculated the average age of cigarette smokers based on adult cohorts reported in each GATS. We then used WHO life tables to identify remaining life expectancy (*L_ij_*) by sex (*i*) and age (*j*), with the latter being the average age of current adult smokers according to GATS.

We used GATS data on average daily cigarette use (*D_i_*) to calculate average annual cigarette consumption (*A_i_*) in each country. We multiplied this volume or quantity measure of consumption by the median price of cigarettes (*P_i_*) from GATS to arrive at an average annual cigarette expenditure (*E_i_*):



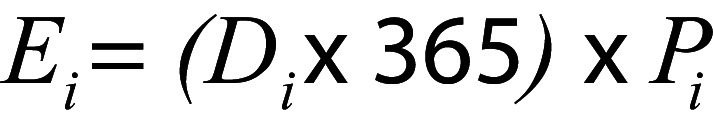

(1)


To establish 2020 as a common benchmark year for each country in the subsequent estimation of life-course spending, we scaled up median prices (*P_i_*) to 2020 levels using the retail price (including taxes) of the most popular brand of cigarette in each country between 2012 and 2022, as reported in the WHO Report on the Global Tobacco Epidemic.^26^ We then used official international exchange rates for July 2020, also published in the report, to convert cigarette prices and expenditure from local currency into both United States dollars (US$) and international dollars or purchasing power parity (PPP) terms.

Finally, we multiplied the average annual cigarette expenditure (*E_i_*) by the respective life expectancies (*L_ij_*) to obtain the life-course cigarette expenditure (*LC_ij_*). As per best practice in health economic evaluations, we converted these life-course expenditures into net present value dollar terms using a social discount rate of 3% plus inflation of roughly 6% per annum:^31^



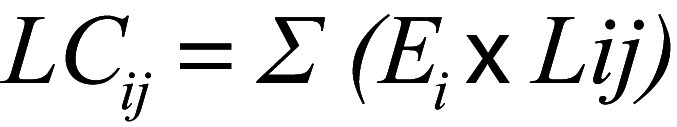

(2)


Our main methodological dilemma was the treatment of cigarette prices over time, as this is a function of the underlying pricing strategy of the industry as well as taxation as a discrete policy intervention. However, in our main analysis we apply a simple cost-plus approach whereby median cigarette prices increase over time in line with inflation. In other words, we assume no new or additional tax policy interventions in these countries. 

## Results

Table 1 shows the primary data extracted from each GATS report for the 15 low- and middle-income countries included in this sample. We observed that the average age of cigarette smokers ranged from 35 years in Senegal to 47 years in India, with an overall average age of 40 years. Cigarette consumption averaged 11 sticks per day (3959 sticks per year). The expenditure on cigarettes by smokers was equivalent to 7.2% of annual average earnings across these countries. We noted that this indicator was highest in Indonesia (with an average cigarette expenditure of 11.9% of average earnings), followed by several countries within the WHO African Region, namely Botswana (9.9%), Ethiopia (9.9%) and the United Republic of Tanzania (9.5%). Cigarette expenditure was lowest relative to annual earnings in Viet Nam (3.9%), Kazakhstan (5.1%), the Russian Federation (5.4%) and China (5.8%); it is unsurprising that just 8.0% (1550/19 376) of the respondents to China’s GATS cited financial pressure as a reason to quit.^25^

**Table 1:** Global Adult Tobacco Survey data on cigarette consumption in 15 low- and middle-income countries, and average annual earnings from ILOSTAT

Country	WHO region	Country income level	Survey year	Average age, years	Daily use, sticks	Average price per pack,^a^ local currency units	Annual expenditure on cigarettes, local currency units	Average annual earnings, local currency units^b^	% of income spent on cigarettes
Bangladesh	South-East Asia	Lower-middle	2017	40	8.1	59.2	8 709	148 360	5.9
Botswana	African	Upper-middle	2017	37	7.8	42.9	6 118	61 906	9.9
China	Western Pacific	Upper-middle	2018	45	16.0	9.8	2 866	49 575	5.8
Ethiopia	African	Low	2016	40	8.9	15.0	2 448	24 734	9.9
India	South-East Asia	Lower-middle	2017	47	6.9	112.4	14 099	188 148	7.5
Indonesia^c^	South-East Asia	Lower-middle	2021	37	7.6	25 921.0	3 585 405	30 138 097	11.9
Kazakhstan	European	Upper-middle	2019	43	15.4	408.8	114 873	2 241 780	5.1
Mexico	Americas	Upper-middle	2015	37	7.7	41.1	5 767	69 561	8.3
Philippines	Western Pacific	Lower-middle	2015	38	10.9	48.0	9 594	137 970	7.0
Russian Federation	European	Upper-middle	2016	41	16.2	80.2	23 646	440 508	5.4
Senegal	African	Lower-middle	2015	35	9.4	549.3	94 276	1 331 813	7.1
South Africa	African	Upper-middle	2021	40	8.6	23.8	3 725	95 764	3.9
Ukraine	European	Lower-middle	2017	41	17.0	17.5	5 428	85 248	6.4
United Republic of Tanzania	African	Lower-middle	2018	39	8.5	2 851.0	442 082	4 629 819	9.5
Viet Nam	Western Pacific	Lower-middle	2015	40	13.7	9 584.0	2 402 482	61 218 477	3.9
**Average**	**NA**	**NA**	**NA**	**40**	**10.8**	**NA**	**NA**	**NA**	**7.2**

NA: not applicable.

^a^ Based on 20 cigarettes per pack.

^b^ ILOSTAT average earnings data by country.^29^

^c^ In accordance with resolution WHA78.25 (2025), Indonesia was reassigned to the WHO Western Pacific Region as of 27 May 2025.

### Cost and prevalence

**Fig. 1:**
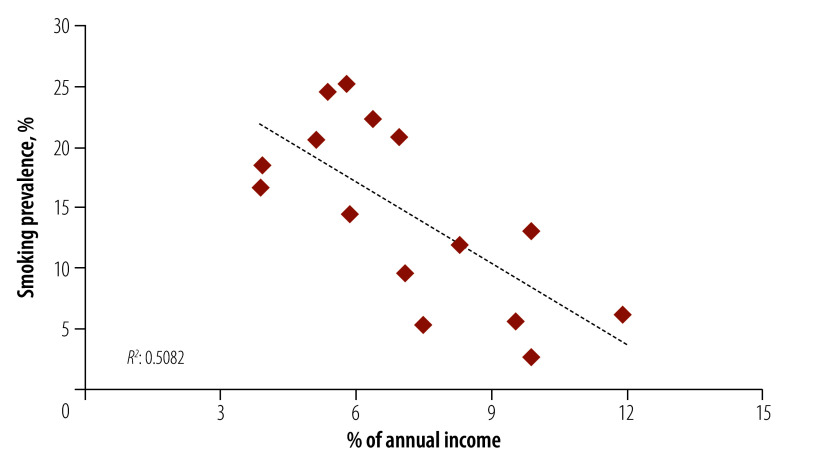
Correlation between smoking prevalence and affordability of cigarettes for 15 low- and middle-income countries in 2020

Fig. 1 highlights the broader relationship between financial cost (measured here as the percentage of annual income spent on cigarettes) and the prevalence of smoking among adults: smoking prevalence is highest in the countries where the financial cost of cigarette consumption is the lowest, and vice versa.

### Life-course consumption

Table 2 shows the life-course consumption of young adults who initiate smoking when aged 15 years, as well as the median price of a pack of 20 cigarettes in US$ for 2020. The average life expectancy of young adults at the age of 15 years is a further 55 years. The total number of cigarettes consumed by smokers over their life-course would therefore average 217 752 sticks. A smoker who started using tobacco at the age of 15 years will spend an average of US$ 8481 on cigarettes in net present value terms over their typical life-course, which is more than twice the current average annual earnings of workers in these countries.

**Table 2:** Life-course expenditure for young adults who started smoking at the age of 15 years (2020 median prices and currency exchange rates)

Country	Average price, US$ per pack	Average price, PPP per pack	Cohort life expectancy, years^a^	Life-course consumption, sticks^b^	Life-course expenditure, US$^c^	Life-course expenditure, PPP^c^	Equivalent income, years
Bangladesh	1.0	2.7	59	173 537	4 403	11 727	1.9
Botswana	4.1	9.9	51	145 372	15 933	38 910	2.9
China	1.5	2.5	61	356 939	13 314	29 914	1.6
Ethiopia	1.1	3.3	57	186 070	5 418	15 816	3.8
India	1.6	5.6	56	140 451	5 913	20 108	2.2
Indonesia	1.7	5.2	55	152 154	6 548	20 522	3.1
Kazakhstan	1.1	3.2	54	303 452	8 550	25 454	1.4
Mexico	2.7	6.1	55	154 281	10 682	24 533	2.8
Philippines	1.7	4.3	54	215 714	9 631	24 288	2.6
Russian Federation	1.6	4.6	55	324 266	13 825	38 905	1.7
Senegal	1.4	3.2	55	188 793	6 795	15 652	2.7
South Africa	1.4	3.3	51	159 802	6 015	14 411	1.1
Ukraine	1.2	4.2	55	341 497	10 463	37 510	2.1
United Republic of Tanzania	1.4	3.7	55	170 584	6 268	16 454	2.8
Viet Nam	0.5	1.5	58	290 775	3 461	10 705	1.0
**Average**	**1.6**	**4.2**	**55**	**217 752**	**8 481**	**22 454**	**2.2**

PPP: purchasing power parity; US$: United States dollars.

^a^ Remaining life expectancy at the age of 15 years.

^b^ Calculated from daily consumption (Table 1) multiplied by 365 multiplied by life expectancy at the age of 15 years.

^c^ Net present value based on the average price of a pack of 20 cigarettes, future inflation and a 3% social discount rate.

### Avoidable expenditure

Table 3 shows the predicted average consumption of adult smokers over the remaining life-course. These results also represent the amount of consumption and expenditure that can be avoided (or saved) by adult smokers through cessation. The remaining life expectancy for adults at the average age of smokers in these countries is a further 34 years. On average, smokers can avoid consuming a further 134 611 cigarettes by quitting. In monetary terms, smokers can avoid an average of US$ 6612 in net present value terms in spending on cigarettes over their remaining life-course by quitting, an amount that is 1.8 times the current average earnings of workers in these countries.

**Table 3:** Life-course expenditure avoided by existing smokers who quit (2020 median prices and currency exchange rates)

Country	Average price, US$ per pack	Average price, PPP per pack	Cohort life expectancy, years^a^	Remaining life-course consumption, sticks^b^	Avoidable life-course expenditure^c^		
					US$	PPP	Equivalent income, years
Bangladesh	1.0	2.7	36	105 887	3 457	9 207	1.5
Botswana	4.1	9.9	33	94 064	12 633	30 852	2.3
China	1.5	2.5	36	210 652	10 320	16 986	1.2
Ethiopia	1.1	2.3	34	110 989	4 170	12 173	2.9
India	1.6	5.6	28	70 225	4 056	13 794	1.5
Indonesia	1.7	5.2	36	99 592	5 290	16 850	2.5
Kazakhstan	1.1	3.2	31	174 204	6 360	18 859	1.0
Mexico	2.7	6.1	37	103 789	8 767	20 136	2.3
Philippines	1.7	4.3	35	139 814	7 714	19 453	2.1
Russian Federation	1.6	4.6	32	188 664	10 408	29 288	1.2
Senegal	1.4	3.2	37	127 006	5 578	12 847	2.2
South Africa	1.4	3.3	33	103 401	4 769	11 426	0.8
Ukraine	1.2	4.2	32	198 689	7 876	28 328	1.6
United Republic of Tanzania	1.4	3.7	37	114 756	5 144	13 505	2.3
Viet Nam	0.5	1.5	34	170 454	2 645	8 182	0.7
**Average**	**1.6**	**4.2**	**34**	**134 611**	**6 612**	**17 435**	**1.8**

PPP: purchasing power parity; US$: United States dollars.

^a^ Remaining life expectancy at average age of current smokers.

^b^ Calculated from daily consumption (Table 1) multiplied by 365 multiplied by remaining life expectancy of current adult smokers.

^c^ Net present value based on the average price of a pack of 20 cigarettes, future inflation and a 3% social discount rate.

**Fig. 2:**
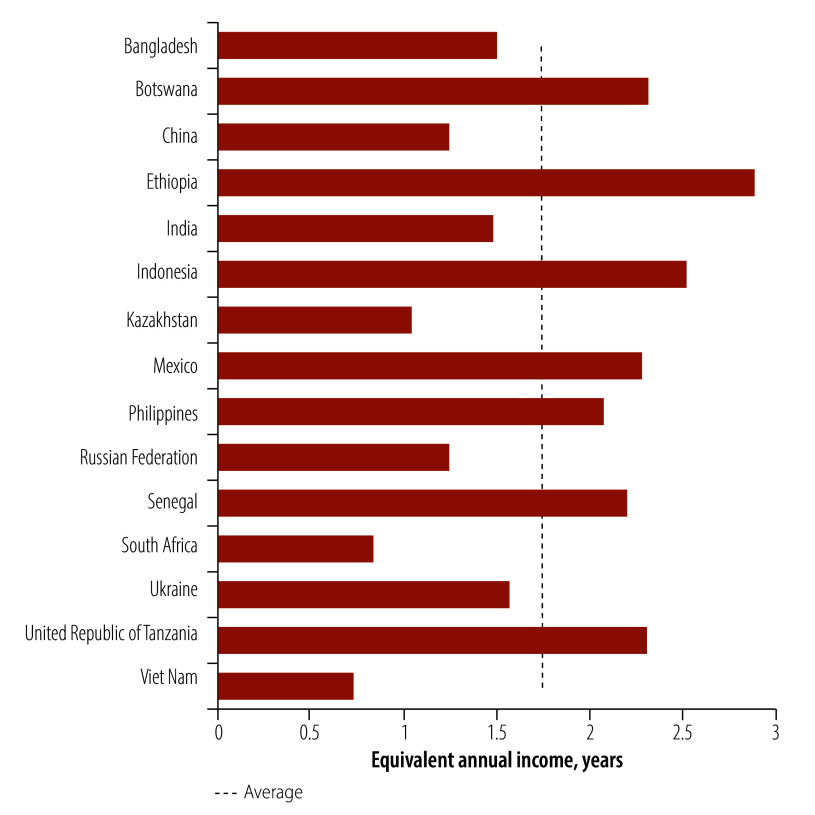
Years of equivalent current annual income saved by current smokers during their remaining life-course through cessation in 2020

Smokers in Botswana can avoid the highest expenditure on cigarettes by quitting, saving US$ 12 633 in net present value terms, an amount equivalent to 2.3 times the current annual earnings in 2020. As shown in Fig. 2, adult smokers in Botswana, Ethiopia, Indonesia, Mexico, the Philippines, Senegal and the United Republic of Tanzania can also avoid expenditure of more than twice the level of current average earnings. These everyday financial savings are in addition to the potential health-care costs and income losses that smokers can avoid by quitting tobacco use. Indeed, much of the harm from smoking can still be reversed through quitting even later in life. For example, a smoker who successfully quits at the age of 40 years (i.e. the average age of adult smokers in our sample) can gain a further 9 years of life expectancy, and a smoker who quits at the age of 60 years can gain a further 3 years of life expectancy.^36^

## Discussion

In calculating the high cost of purchasing cigarettes across the full life-course of smokers, we have highlighted the financial savings or benefits available from cessation. Our analysis assumed no new tobacco control interventions, meaning that cigarettes will become more affordable over time. The WHO Report on the Global Tobacco Epidemic showed that cigarettes have become less affordable in only about one third of all countries, and have actually become more affordable in approximately one sixth of all countries.^26^ It is clear that higher taxes are needed to address market failures in the price of cigarettes and other tobacco products. Higher tobacco taxes will help prevent young adults taking up smoking, as well as provide a strong financial incentive for smokers to quit.

Our finding showing that smoking prevalence was negatively correlated with the percentage of annual income spent on cigarettes is very similar to the concept of affordability, which is measured in tobacco economics as the percentage of gross domestic product (GDP) required to purchase 100 packs of cigarettes.^26^ This concept was first introduced in 2004, when a study with a sample of 42 low- and middle-income countries found that a 1% decrease in affordability would lead to a decrease of 0.53% (95% confidence interval, CI: −0.49% to −0.57%) in per capita cigarette consumption.^32^ More recently, a study assessed the relationship between affordability and smoking participation or prevalence in eight WHO African Region countries, finding that a 1% decrease in affordability leads to a decrease of 0.25% (95% CI: −0.41% to −0.08%) in participation.^33^ Although we do not apply the same level of statistical rigour to the data when assessing the correlation between smoking prevalence and affordability of cigarettes, the same essential pattern is clear.

We also observe strong similarities to the price–prevalence elasticity of demand, which measures the relationship between cigarette prices and smoking participation. International evidence suggests that the price–prevalence elasticity of demand is centred around −0.2 in low- and middle-income countries.^4^^,^^34^^,^^35^ There is evidence of this effect in at least one country from our sample. Several GATS studies have been conducted in the population of the Philippines over the past decade, which show that the prevalence of smoking has fallen by over one third in relative terms, or from 27.0% (16.5 million/61.3 million) of adults in 2009 to 17.4% (13.5 million/77.6 million) of adults in 2021.^25^ The Philippine government implemented a roadmap of significant tobacco tax reforms over this timeframe as part of the 2012 Sin Tax Reform law, leading to the median cigarette price as measured by GATS to increase by 154% in inflation-adjusted terms between 2009 and 2021.^25^ Such changes imply a price–prevalence elasticity of roughly −0.23 (−0.36/1.54), with this example being very close to international findings.

Interestingly, the 2015 GATS study for the Philippines found that two thirds of respondents had reported attempting to quit because of high prices.^25^ Other GATS studies highlight people’s reaction to potential tax and price increases: almost one half of respondents in Ukraine reported that they would quit or reduce use compared with just one fifth who reported that their consumption would not change.^25^

The evidence that higher cigarette prices will encourage smokers to quit is consistent with the results from more sophisticated discrete-choice experiments. Although this type of research is still relatively new in the field of tobacco control, available studies highlight that monetary considerations are highly influential in shaping the responses of smokers, and that cigarette prices are positively associated with the likelihood of attempting to quit.^37^ One of the earliest such experimental studies emphasized the importance of the magnitude of the price increase.^38^ Separate discrete-choice experiments in Indonesia, the Republic of Korea and Viet Nam suggested that cigarette prices need to be 2–3 times higher for respondents to quit.^39^^–^^41^

Although it is not possible without more data to model the overall average outcome of different behavioural pathways, such as quitting and cutting down, we can certainly highlight that higher taxes will make the life-course benefits of cessation even greater. For example, if median cigarette prices were to double compared with the baseline in our main analysis, then the average life-course savings of those who quit would increase to US$ 13 225 in net present value terms, or to around 3.5 times the current annual earnings of workers in these countries. Beyond this simple example, people’s expectations about prices in the future are certainly important, with large tax increases encouraging the cessation of smoking.

It is also important to understand that taxation works most effectively in combination with other MPOWER policies also aimed at tackling market failures, such as health warnings or smoke-free laws. Indeed, the WHO FCTC and MPOWER package of policies for country-level implementation also reflects behaviouralist theory, with many being proverbial nudge interventions.^42^

The importance of cessation support has come into sharper focus since the COVID-19 pandemic. This support includes the new WHO cessation consortium, which aims to support the scale-up services at country level for treating tobacco dependence.^43^ Such services include the emergence of digital mCessation applications to provide clear information about the health risks of smoking and to support attempts to quit by smokers.^44^ Evidence suggests that mCessation apps may increase the likelihood of cessation success by 50–60%.^45^^,^^46^

The consortium also aims to expand access to nicotine replacement therapy, varenicline and bupropion. The 2021 WHO global investment case for cessation highlights the strong economic return to countries from investing in such pharmacological interventions.^47^ Interestingly, the WHO global investment case for cessation estimated an average cost per quitter of US$ 1553 for such interventions in developing country settings.^47^ Even when assuming these costs were met out of pocket by smokers, our study suggests a return of 4:1 for those who quit using such interventions simply as a result of avoided cigarette expenditure. When factoring in lower overall health-care expenditure and the healthy life-years gained from cessation, the returns could be even higher.

Our study has several limitations, including that some surveys are now nearly a decade old. In addition, our analysis is not very nuanced given the wide range of tobacco products and product prices on the market. The availability of very cheap or low-priced cigarette brands presents an ongoing challenge in many countries since they encourage uptake by young adults. The emergence of new or novel tobacco products also presents a significant new challenge, especially since flavours are a key reason young adults try them. While taxation has a key role in curbing the demand for all tobacco products, consideration should also be given to prohibiting at least the most harmful product forms or variants that are clearly designed to entice young adults.

The level of price and affordability of cigarettes are important determinants of cigarette use and consumption, and tax policies can have a large effect on consumers, especially young adults. Overall, these costs (and benefits of cessation) will only increase over time as governments around the world continue to raise taxes to address the market failures inherent within the tobacco market.
